# Non-myogenic tumors display altered expression of dystrophin (DMD) and a high frequency of genetic alterations

**DOI:** 10.18632/oncotarget.10426

**Published:** 2016-07-06

**Authors:** Leonela N. Luce, Mercedes Abbate, Javier Cotignola, Florencia Giliberto

**Affiliations:** ^1^ INIGEM, CONICET / Cátedra de Genética y Biología Molecular, Facultad de Farmacia y Bioquímica, Universidad de Buenos Aires, Argentina; ^2^ IQUIBICEN, CONICET / Departamento de Química Biológica, Facultad de Ciencias Exactas y Naturales, Universidad de Buenos Aires, Argentina

**Keywords:** DMD, dystrophin, gene expression, genetic alteration, survival

## Abstract

*DMD* gene mutations have been associated with the development of Dystrophinopathies. Interestingly, it has been recently reported that DMD is involved in the development and progression of myogenic tumors, assigning DMD a tumor suppressor activity in these types of cancer. However, there are only few reports that analyze DMD in non-myogenic tumors. Our study was designed to examine *DMD* expression and genetic alterations in non-myogenic tumors using public repositories. We also evaluated the overall survival of patients with and without *DMD* mutations. We studied 59 gene expression microarrays (GEO database) and RNAseq (cBioPortal) datasets that included 9817 human samples. We found reduced *DMD* expression in 15/27 (56%) pairwise comparisons performed (Fold-Change (FC) ≤ 0.70; p-value range = 0.04-1.5×10^−20^). The analysis of RNAseq studies revealed a median frequency of *DMD* genetic alterations of 3.4%, higher or similar to other well-known tumor suppressor genes. In addition, we observed significant poorer overall survival for patients with *DMD* mutations. The analyses of paired tumor/normal tissues showed that the majority of tumor specimens had lower *DMD* expression compared to their normal adjacent counterpart. Interestingly, statistical significant over-expression of *DMD* was found in 6/27 studies (FC ≥ 1.4; p-value range = 0.03-3.4×10^−15^). These results support that *DMD* expression and genetic alterations are frequent and relevant in non-myogenic tumors. The study and validation of DMD as a new player in tumor development and as a new prognostic factor for tumor progression and survival are warranted.

## INTRODUCTION

*DMD* gene (Xp21.2-p21.1, OMIM #300377) spans 2.4 Mb. It contains 79 exons, eight different promoters that regulate tissue-specific expression, multiple alternative splicing and polyadenylation sites that give rise to, at least, 15 different DMD isoforms. Historically, mutations in *DMD* are associated with the development of Dystrophinopathies: Duchenne Muscular Dystrophy (DMD), Becker Muscular Dystrophy (BMD) and X-linked Dilated Cardiomyopathy (XLDC).

The DMD skeletal muscle isoform is the best characterized and its main function is well-known. This protein interacts with actin fibers from the cytoskeleton and with the Dystrophin-Associated Glycoproteins complex (DAG). These interactions link the cytoplasm to the extracellular matrix, and play a major role in maintaining sarcolemma membrane stability and organization, cell signaling, regulating the intracellular calcium and muscle homeostasis [1]. However, the function of the other dystrophin isoforms remains to be fully elucidated.

Interestingly, it has been reported that DMD and its partners (e.g. dystroglycan, dysferlin, calpain-3, Large) are involved in tumor development and progression [2–6]. *DMD* was found frequently under-expressed in melanoma cell lines, and this reduced expression was due to gene deletions [3]. In addition, *in vitro* down-regulation of *DMD* enhanced melanoma cells migration and invasion [3].

*In vivo* studies using mouse models for muscular dystrophies showed a high frequency of development of skeletal-muscle associated tumors such as myosarcomas, liposarcomas and fibrosarcomas [4]. This study also reported non-random genetic abnormalities in skeletal muscles from the animal models and from patients with muscular dystrophies [4]. More recently, Wang *et al*. demonstrated that *DMD* intragenic somatic deletions were common in myogenic tumors and were associated with the progression to high-grade lethal sarcomas [6]. *DMD* deletions were also more frequent in myogenic sarcomas compared to non-myogenic sarcomas (25/40 *vs* 0/58, respectively) and non-sarcoma tumors (25/40 *vs* 39/866) [6]. The study also showed that restored expression of a mini-DMD construct inhibited myogenic sarcoma cell migration, invasion, anchorage independence and invadopodia formation [6].

Although the abovementioned publications showed that *DMD* deletions are infrequent in non-myogenic tumors, *DMD* mRNA levels were not fully analyzed in different tumor types. Therefore, the aim of this study was to evaluate *DMD* expression and genetic alterations in non-myogenic tumors. To accomplish this, we designed a bioinformatic study using data from public repositories.

## RESULTS

### DMD expression is altered in the majority of the analyzed tumors

We analyzed 16 different types of non-myogenic tumors that included 1765 human samples. Table 1 summarizes the sample series (GSEs) included, and the 27 pairwise comparisons performed to study differential *DMD* expression.

**Table 1:** *DMD* expression analyses using GEO repository data

Sample series	Affymetrix Platform	Probe set	Pairwise comparisons^a^	FC^b^	p-value	Percentile^c^	Ref.
***DMD* under-expression**							
GSE3189	HG-U133	203881_s_at	Benign Nevi (18) / Normal Skin (7)	0.56	6.18×10^−3^	16.6%	[19]
			Melanoma (45) / Benign Nevi (18)	0.36	3.62×10^−10^	5.6%	
			Melanoma (45) / Normal Skin (7)	0.20	5.83×10^−10^	7.4%	
GSE6919	HG-U95C	40488_at	Primary Prostate Tumor (65) / Normal Prostate Tissue (81)	0.60	7.27×10^−9^	0.3%	[20, 21]
			Metastatic Prostate Tumor (25) / Primary Prostate Tumor (65)	0.48	6.55×10^−9^	7.4%	
			Metastatic Prostate Tumor (25) / Normal Prostate Tissue (81)	0.28	1.49×10^−20^	1.4%	
GSE10072	HG-U133	203881_s_at	Lung Adenocarcinoma (58) / Normal Lung Tissue (49)	0.51	2.30×10^−11^	8.0%	[22]
GSE19804	HG-U133	203881_s_at	NSCLC (60) / Adjacent Normal Lung Tissue (60)	0.63	2.79×10^−4^	5.4%	[23]
GSE43458	HuGene-1_0-st	8171921	Lung Adenocarcinoma (80) / Normal Lung Tissue (30)	0.56	8.03×10^−10^	5.5%	[24]
GSE10797	HG-U133	203881_s_at	Tumor Breast Epithelium (28) / Normal Breast Epithelium (5)	0.16	2.05×10^−4^	0.4%	[25]
GSE36295	HuGene-1_0-st	8171921	Breast Cancer Tissue (45) / Normal Breast Tissue (5)	0.29	8.57×10^−5^	2.6%	[26]
GSE15471	HG-U133	203881_s_at	Pancreatic Ductal Adenocarcinoma (36) / Normal Pancreatic Tissue (36)	0.42	2.31×10^−4^	40.8%	[27]
GSE44076	HG-U219	11722991_a_at	Colon tumor (98) / Adjacent paired normal mucosa (98)	0.49	2.01×10^−11^	31.0%	[28, 29]
GSE50161	HG-U133	203881_s_at	Medulloblastoma (22) / Non-tumor brain (13)	0.62	0.036	41.8%	[30]
GSE12453	HG-U133	203881_s_at	Lymphomas (42) / Normal centroblasts and centrocytes (9)	0.17	3.57×10^−8^	3.3%	[31, 32]
***DMD* over-expression**							
GSE48558	HuGene-1_0-st	8171921	Primary T-ALL (13) / Normal T lymphocytes (17)	1.57	4.47×10^−4^	13.3%	[33]
GSE22529	HG-U133	203881_s_at	CLL (41) / Normal B lymphocytes (11)	4.40	2.06×10^−4^	3.3%	[34]
GSE31048	HG-U133	203881_s_at	B-CLL (179) / Normal B lymphocytes (24)	6.12	4.04×10^−6^	8.2%	[35]
GSE53757	HG-U133	203881_s_at	Renal cell carcinoma (72) / Normal kidney simple (72)	1.91	3.39×10^−15^	15.6%	[36]
GSE50161	HG-U133	203881_s_at	Ependymoma (46) / Non-tumor brain (13)	1.57	0.033	49.5%	[30]
			Astrocytoma (15) / Non-tumor brain (13)	1.71	0.027	49.4%	
**Non-significant *DMD* expression changes^d^**							
GSE48558	HuGene-1_0-st	8171921	Primary B-ALL (27) / Normal B lymphocytes (11)	0.84	0.325	63.3%	[33]
			Primary AML (18) / Normal mielocytes (18)	0.97	0.507	83.8%	
GSE50161	HG-U133	203881_s_at	Glioblastoma (34) vs Non-tumor brain (13)	0.95	0.812	63.4%	[30]
GSE47927	HuGene-1_0-st	8171921	CML (48) / Normal patient sample (15)	1.27	0.225	44.8%	[33]
GSE9476	HG-U133	203881_s_at	AML Leukemic blasts (26) / Normal hematopoietic cells, bone marrow (18)	1.03	0.363	44.8%	[37]
			AML Leukemic blasts (26) / Normal hematopoietic cells, peripheral blood (20)	1.01	0.749	60.8%	

a The numbers in parentheses indicate the number of samples analyzed

b FC (Fold-Change) = 2^LogFC

c percentile in which DMD lay when all genes are in ascendant order of p-value

d 0.70<FC<1.40, or p>0.05

Abbreviations: ALL, acute lymphoid leukemia; AML, acute myeloid leukemia; CLL, chronic lymphoid leukemia; CML, chronic myeloid leukemia; NSCLC, non-small cell lung carcinoma.

Since the dystrophin is encoded in the X chromosome, we first analyzed the sample series that included information on gender to seek whether there was a gender-specific expression. As expected, due to X chromosome inactivation in females, there was no difference on dystrophin expression between genders (Supplementary Table S1).

Furthermore, in order to validate our analyses with previous reported findings showing *DMD* deletions on myogenic tumors [6], we analyzed studies that performed gene expression microarrays on leiomyosarcomas and gastrointestinal stromal tumors (GIST). Even though the number of samples was limited for this analysis, a non-significant lower expression was detected in the tumors (Supplementary Table S2). We also found lower *DMD* expression in melanoma compared to normal skin. These results supported the previous reports [3] and validated our analyses.

**Figure 1:**
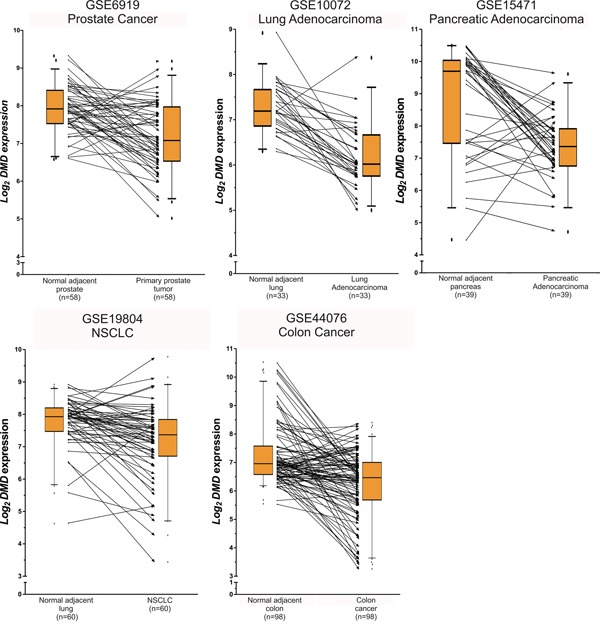
Paired analyses of *DMD* expression between normal and tumor tissues The figure shows the log_2_ *DMD* expression for the series matrixes that included tumor and normal adjacent tissues. The boxplots indicate the median and the 25-75 percentile range, and the whiskers show the 5-95% percentiles. The lines between the boxplots conect the paired normal/tumor samples to represent *DMD* expression changes between both biospecimens. Even tought, for some tumor specimens *DMD* is upregulated, it is under-expressed in most tumor tissues compared to the normal counterpart.

Our study showed that the expression of *DMD* was reduced in 15/27 comparisons. In 60% (9/15) the expression was strongly reduced (FC≤0.50), and in 40% (6/15) was moderate diminished (0.50<FC≤0.70). The analyses of paired tumor/normal tissues showed that the majority of tumor specimens had lower *DMD* expression compared to the normal adjacent tissue (Figure 1).

In addition, we detected an increased expression of *DMD* in 6/27 comparisons. In 33% (2/6) *DMD* expression was strongly augmented (FC≥2.00), and it was moderate increased (1.40≤FC<2.00) in 67% (4/6). We did not find significant changes (0.70<FC<1.40, or p>0.05) in 6/27 comparisons (Table 1).

Finally, we ranked *DMD* within the complete list of genes included in the microarrays. Of the 21 statistically significant comparisons, *DMD* ranked within the top 10% genes more differentially expressed in 13 comparisons (Table 1). To ensure that *DMD* altered expression is not a random event, we evaluated the percentage of the total number of genes that do not change their expression between normal and tumor samples. We found that 75% (median) of all genes (range: 44-99%) did not show statistical significant differential expression, supporting that *DMD* altered expression is not due to chance. We also found that DMD was in a lower percentile (higher rank) compared with other tumor suppressors. DMD ranked higher than BRCA1 in 67% of all comparisons made, higher that BRCA2 in 76%, higher than RB1 in 52%, and higher that PTEN in 62%. These results show that DMD deregulation in tumor tissues is greater compared to other genes.

### DMD mutations are frequent in tumor tissues

**Figure 2:**
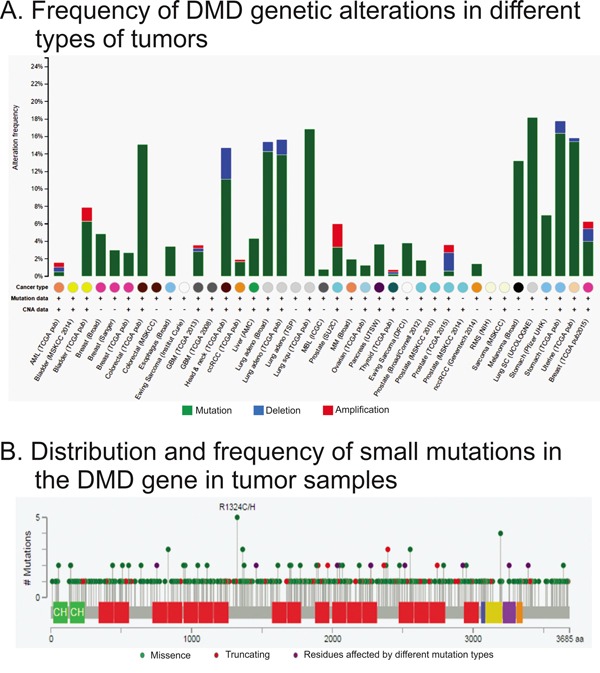
Analysis of genetic alterations in *DMD* using cBioPortal data The figure was ploted using cBioPortal website and depicts the frequency **A.** type and location of *DMD* **B.** genetic alterations found by RNAseq. Panel A shows the frequency and type of mutations for each study analyzed. The x-axis shows the types of cancer (color coded), availability of mutation and copy number variation data, and the study abbreviation. Panle B displays the localization and frequency of all small mutations.

We analyzed the type and frequency of mutations in the *DMD* gene reported in the cBioPortal database. This analysis included NGS data from 8052 samples of different tumor types. We found that the majority of *DMD* genetic alterations corresponded to small mutations, and a very low frequency of gene deletions or amplifications (Figure 2A). The occurrence of *DMD* alterations varied across the studies/tumor types, but it was consistent for some tumor types such as breast and lung cancers (Figure 2A). The median frequency of *DMD* alterations was 3.4%. This frequency was higher than the median gene alteration frequencies for other well-known tumor suppressor genes for the same studies (BRCA1: 1.6%; BRCA2: 2.8%; PTEN: 3.0%; RB1: 3.9%). Moreover, the median frequency of *DMD* alterations in sporadic breast cancer was higher than the median frequency for BRCA genes in the same tumors (3.95% *vs* 1.95% and 3.40% for *DMD*, *BRCA1* and *BRCA2*, respectively). Interestingly, *DMD* alterations were not found in rabdomyosarcomas.

The cBioPortal tool also allowed us to study the type and localization of the small mutations. We observed that there were not hot-spots (Figure 2B). We also analyzed the type of mutation reported, and we found that 15.7% should encode a truncated form of the dystrophin (Table 2). Among the missense mutations there was a 70.3% that were predicted to have a low or medium impact on the protein function (Table 2).

**Table 2:** *DMD* mutations analysis from cBioPortal

	Type of mutation			
	missense	non-sense	In/Del with reading-frame shift	in splicing sites
	413 (84.3%)	34 (6.9%)	23 (4.7%)	20 (4.1%)
***In silico* prediction of impact on protein function**				
neutral	102 (24.7%)	na	na	na
low	151 (36.6%)	na	na	na
medium	139 (33.7%)	na	na	na
no data	21 (5.1%)	na	na	na

na: not applicable.

### Patients with DMD alterations have poorer overall survival

**Figure 3:**
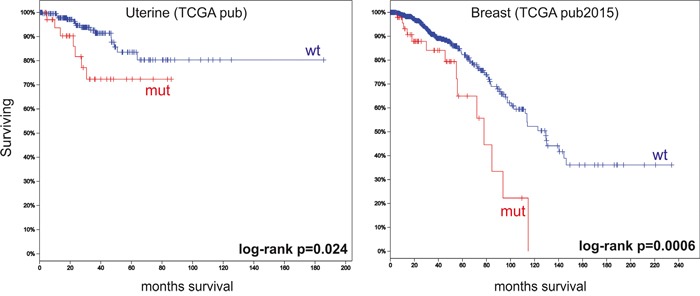
Overall survival analyses using cBioPortal data The figure depicts Kaplan–Meier curves for overall survival stratified by wild-type *DMD* (wt) or mutated *DMD* (mut). The figure shows only the two studies that revealed significant differences between the two groups. Marks denote censored patients. Patients with having *DMD* mutations have poorer overall survival.

Finally, we conducted a survival analysis using cBioPortal. Follow-up data was only available for 11 studies. We observed that patients with genetic alterations in *DMD* had significantly poorer OS compared to patients with wild-type *DMD* in 2/11 studies (Figure 3).

## DISCUSSION

Historically, germline *DMD* mutations have been associated with the development of Muscular Dystrophies. However, the involvement of *DMD* gene in tumorigenesis is emerging. This study aimed to analyze *DMD* gene expression and mutation frequency in non-myogenic tumors using microarray and RNAseq data from public repositories. In addition, this type of bioinformatics study highlights the importance of public genetic repositories that allow analyzing data beyond the original aim of the study.

Previous reports showed a high frequency of *DMD* intragenic deletions that were associated with the progression of myogenic tumors [4–6, 15]. Similar results were found in melanoma cell lines [3]. These reports were mainly based on experiments on cell lines, mouse models and in a limited number of human samples, and were focused on seeking *DMD* intragenic deletions. Therefore, the relevance of our study relied on the analysis of over 9.000 human samples and on the evaluation of *DMD* gene expression, mutation frequency and overall survival.

In concordance to the previous reports suggesting the tumor suppressor role of DMD, we found that *DMD* expression was decreased in the majority of the analyzed tumors compared to the normal tissues. Remarkably, we found that *DMD* expression was decreased in primary prostate tumors and further reduced in metastasis. We also observed that *DMD* expression was diminished in melanoma compared to benign nevi that already showed a reduced expression compared to normal skin samples. These results confirmed a role of DMD in tumorigenesis. The molecular mechanism involved in *DMD* decreased expression remains to be studied.

The analysis of *DMD* genetic alterations revealed a high frequency of gene mutations, which was similar to other well-known tumor suppressor genes (BRCA1, BRCA2, PTEN, RB1). Likewise, the presence of a mutation in *DMD* shortened the overall survival of patients with Uterine Corpus Endometrioid Carcinoma and Breast Invasive Carcinoma. Similar results were published by Stephens *et al.*, who observed shorter survival for patients with upper gastrointestinal cancer and low expression of *DMD* [16].

Interestingly, we also found that *DMD* was over-expressed in leukemias, renal carcinomas, ependymomas and astrocytomas. These results were similar to previous reports where it was shown that *DMD* expression was higher in B-cell Chronic Lymphocytic Leukemia compared to normal B-cells [17]. High *DMD* expression was also associated with poorer overall survival and shorter lymphocyte doubling time [17]. Other studies demonstrated that DMD Dp71 plays a central role in proliferation [6, 18], invasion and migration *in vitro* [18] and suppressed tumor growth in xenograft models [18]. Although the molecular mechanism underlying DMD over-expression and oncogenic activity is unknown, we suspect that it might be related to an increase expression of the Dp71 isoform. These results warrant further studies to investigate the different functions of dystrophin isoforms.

The limitations of our study are that we could not differentiate the expression of different isoforms and that lower mRNA levels might not correlate with lower protein levels.

Overall, our results strengthen the involvement of DMD in carcinogenesis. This links DMD, currently considered to be responsible only for the development of the monogenic orphan dystrophinopathies, to one of the most frequent diseases such as cancer. The study and validation of *DMD* as a new player in tumor development and as a new prognostic factor for tumor progression and survival are warranted.

## MATERIALS AND METHODS

### Gene expression microarray data

#### Sample series

**Figure 4:**
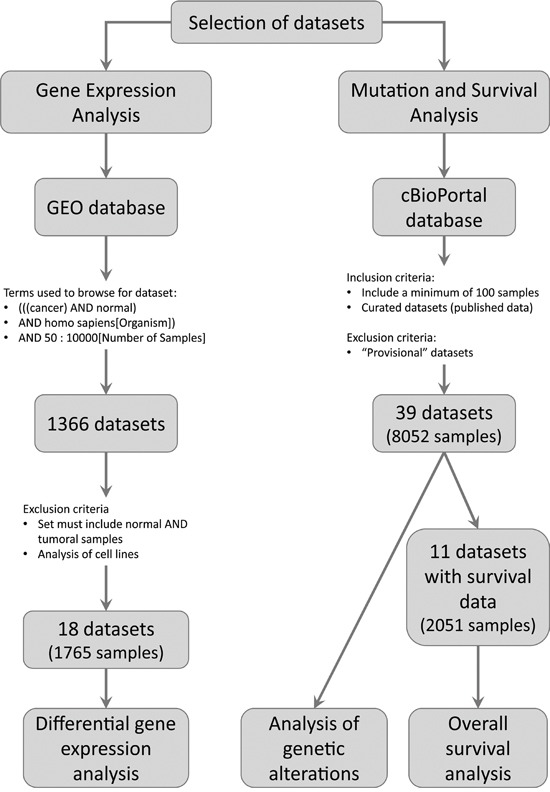
Pipeline used to select the datasets to be analyzed The figure shows the inclusion and exclusion criteria used to select the datasets. In addition, the number of datasets and samples analyzed are depicted.

We used the public repository Gene Expression Omnibus (GEO) from the National Center for Biotechnology Information (NCBI) [7, 8] to browse for gene expression microarrays data. We aimed to identify studies that included normal and tumoral human tissues. The following keywords and expressions were used to browse in the GEO repository: (((cancer) AND normal) AND homo sapiens[Organism]). We only considered the studies that analyzed both normal and tumoral tissues in order to reduce to a minimum the inter-laboratory variability (Figure 4). The series analyzed are listed in Table 1.

Protocol approvals and informed consents were obtained by the authors of the original studies.

### Pre-processing of raw data

We downloaded the raw microarray data and we used the R-based software Bioconductor [9, 10] to perform the quality control and pre-processing of the data. We used the RMA (Robust Multi-array Average) algorithm [11] to correct for background, normalize and log_2_-transform the readings for each sample. We used a principal component analysis to determine whether there was a batch effect within the series analyzed.

### DMD differential expression analyses

To investigate the differential expression between normal and tumoral tissues we used a linear model from the Bioconductor ‘limma’ package [12] to calculate the LogFC (Log_2_-Fold Change (FC) between two sample groups) and the p-values for the comparisons using an umpaired two-tail t-test. We corrected the models for batch effect when needed.

When the raw data were not available in GEO, we used the GEO2R tool at the NCBI web site. GEO2R is a Bioconductor-based online interphase that allows to compare gene expression in different groups of samples. GEO2R calculates the LogFC and the p-values for the comparisons using an unpaired two-tail t-test. This tool does not allow to perform custom quality control, pre-processing of the data or to correct for batch effect.

Because the size and the complexity of the *DMD* gene, most commercial gene expression microarrays are designed to interrogate dystrophin expression using multiple probe sets. For this analysis, we interrogated only the most 3′-mapping probe set that detects all dystrophin isoforms (Table 1). Only the isoform Dp40 is not detected by these probes. Therefore, the changes observed in *DMD* expression could not be linked to any specific isoform.

Since we only tested the differential expression for *DMD*, we considered the unadjusted p-value. We determined that *DMD* was highly differentially expressed when gene expression was, at least, twice (FC≥2.00) or half (FC≤0.50) the expression of the normal counterpart. We defined a moderate change in expression when there was a 50% to 99% increase (1.40≤FC<2.00) or reduction (0.50<FC≤0.70) in *DMD* mRNA levels. In both cases, p-values should also be less than 0.05. Gene expression changes lower than ±50% (0.70<FC<1.40) compared to the normal tissue were not considered significant even if p≤0.05.

### Next-Generation Sequencing (NGS) data

We browsed the public database cBioPortal for Cancer Genomics [13, 14]. This portal collects NGS data from The Cancer Genome Atlas (TCGA) and the International Cancer Genome Consortium (ICGC). We analyzed data from published cancer studies that included a minimum of 100 samples (Figure 4). We also included one study that analyzed 43 rabdomyosarcomas with the goal of comparing our results with previous reports. The studies analyzed are listed in the Supplementary Table S3.

We used the online interphase to determine the frequency of genetic alterations in *DMD* (point mutations and In/Dels) and to study the overall survival (OS) of patients with and without *DMD* genetic alterations. For OS analyses, we only considered the studies with at least 10 patients with genetic alterations. Kaplan-Meier curves stratified by genotype were plotted and the comparisons were tested using the Log-rank test.

Protocol approvals and informed consents were obtained by the authors of the original studies.

## SUPPLEMENTARY TABLES




