# Supplementary material for: Non-myogenic tumors display altered expression of dystrophin (DMD) and a high frequency of genetic alterations
Source: Oncotarget. 2016 Jul 6;8(1):145–55. doi: 10.18632/oncotarget.10426 (PMC5352069; doi:10.18632/oncotarget.10426)
Supplement: Supplementary file 1 [file oncotarget-08-145-s001.pdf]

Non-myogenic tumors display altered expression of dystrophin (DMD) and a high frequency of genetic alterations

SUPPLEMENTARY TABLES

Supplementary Table S1: *DMD* differential expression between Females and Males

Sample Series	probe	Females(normal)/ Males(normal)			Females(tumoral)/ Males(tumoral)			Females(normal+tumoral)/ Males(normal+tumoral)		
		p-value	Log FC	FC	p-value	Log FC	FC	p-value	Log FC	FC
GSE10072	203881_s_at	0.240	0.227	1.170	0.509	0.118	1.085	0.632	0.080	1.057
GSE39716	8171921	0.007*	-0.843	0.557	0.914	-0.029	0.980	0.639	-0.112	0.925
GSE44076	11722991_a_at	0.135	-0.254	0.839	0.268	-0.292	0.817	0.158	-0.217	0.861

*this study included only a limited number of samples (3 normal tissues from Females and Males); therefore, this difference was not considered significant.

Supplementary Table S2: DMD expression in myogenic tumors

Reference Series	Samples analyzed	Probe	FC	p.Value
GSE2719	2 normal tissues (stomach & small intestine) vs. 2 GIST	203881_s_at	0.80	0.721
GSE2719	2 normal tissues (stomach & small intestine) vs. 6 leiomyosarcomas	203881_s_at	2.19	0.222
GSE2685	8 non-tumoral gastric tissues vs. 22 primary human advanced gastric cancer tissues	L35854_at	0.65	0.079
		M18533_at	0.68	0.120
		S81419_at	0.78	0.153
GSE764	4 normal myometrium vs. 9 uterine leiomyosarcoma + 4 extra-uterine leiomyosarcoma	L35854_at	0.51	0.136
		M18533_at	0.46	0.194
		S81419_at	0.80	0.644

Supplementary Table S3: Cancer studies analyzed from cBioPortal

See Supplementary File 1
